# An Intra-tendonous ganglion cyst causing impingement between the anterior cruciate ligament and anterior root of the medial meniscus: a case report

**DOI:** 10.1186/2052-1847-5-22

**Published:** 2013-10-17

**Authors:** Mei Guolong, Gao Zhi, Hu Yong

**Affiliations:** 1 Department of arthroscope, Sichuan orthopeadic Hospital Chengdu, Sichuan 610000, China

**Keywords:** Ganglion cyst, Anterior cruciate ligament, Medial meniscus

## Abstract

**Background:**

There are several reports of symptomatic ganglion cysts near the anterior cruciate ligament (ACL), posterior cruciate ligament (PCL), and lateral and medial meniscus, but symptomatic ganglia arising from the anterior horn of the medial meniscus to the ACL have not been reported. Here we report the arthroscopic resection of a ganglion cyst arising from the anterior horn of the medial meniscus with a meniscal tear to the ACL.

**Case presentation:**

A 43-year-old female presented with a 10-year history of continuous aching pain in the right knee, but without any history of trauma. Clinical examination revealed right-sided knee pain in the medial joint line, exacerbated by end range flexion and extension, a −10°-100° active range of movement, and a −5°-110° passive range of movement。McMurray’s, patellar compression, and compression rotation tests were positive. Magnetic resonance imaging (MRI) and arthroscopic examination revealed a cyst related to the ACL and medial meniscus. Histological examination confirmed the cyst to be a ganglion cyst.

**Conclusions:**

We present a new type of ganglion cyst, this is the first reported case of an ganglion cyst impinged between the ACL and the medial meniscus. It is hoped that this study will provide a better understanding of the condition and lead to better diagnosis and treatment.

## Background

Since Caan first reported, in a 1924 autopsy, a ganglion cyst on the anterior cruciate ligament [1], more and more ganglion cysts around the knee have been reported [2-4], the common sites of cysticlesions of knee joint is the ACL followed by PCL, and then menisci, especially medial meniscus; other rare sites are infrapatellar pad of fat, medial plica and popliteus tendon [5]. A ganglion cyst is often defined as a cyst swelling that is formed of myxiod matrix, which can lead to cystic lesions associated with a joint or tendon sheath [6,7]. No reports have yet appeared concerning a symptomatic ganglion impingement between the ACL and medial meniscus. We report a case of a symptomatic ganglion cyst that originated from the anterior horn of the medial meniscus and extended to the ACL.

## Case presentation

A-43-year-old female presented with a 10-year history of right knee pain of insidious onset. The problem began as an aching pain, had worsened in last 10 days, and was particularly noticeable when standing after squatting and while going up and down the stairs. The patient reported no history of trauma and no previous knee problems. She was 158 cm tall and 64 kg in body weight.

**Figure 1:**
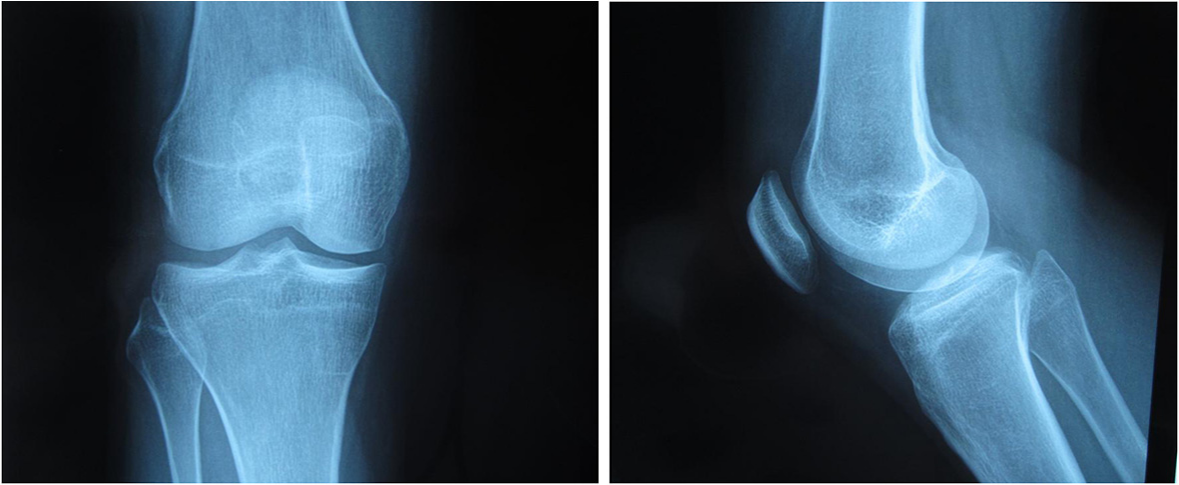
Plain film radiograph of the anterior-posterior and lateral right knee show no abnormalities or loose bodies.

Physical examination revealed right-sided knee pain in the medial joint line, exacerbated by end range flexion and extension, and without swelling or bruising. No mass was palpable around the knee. The active range of movement was −10°-100°, and passive range of movement was −5°-110°. Atrophy of the right quadriceps was present. Anterior and posterior drawer and Lachmann tests were found to be negative, but the McMurray’s test, patellar compression test, and compression rotation test were positive. Plain film radiographs of the knee were performed and displayed no bone abnormalities or loose bodies (Figure 1).

**Figure 2:**
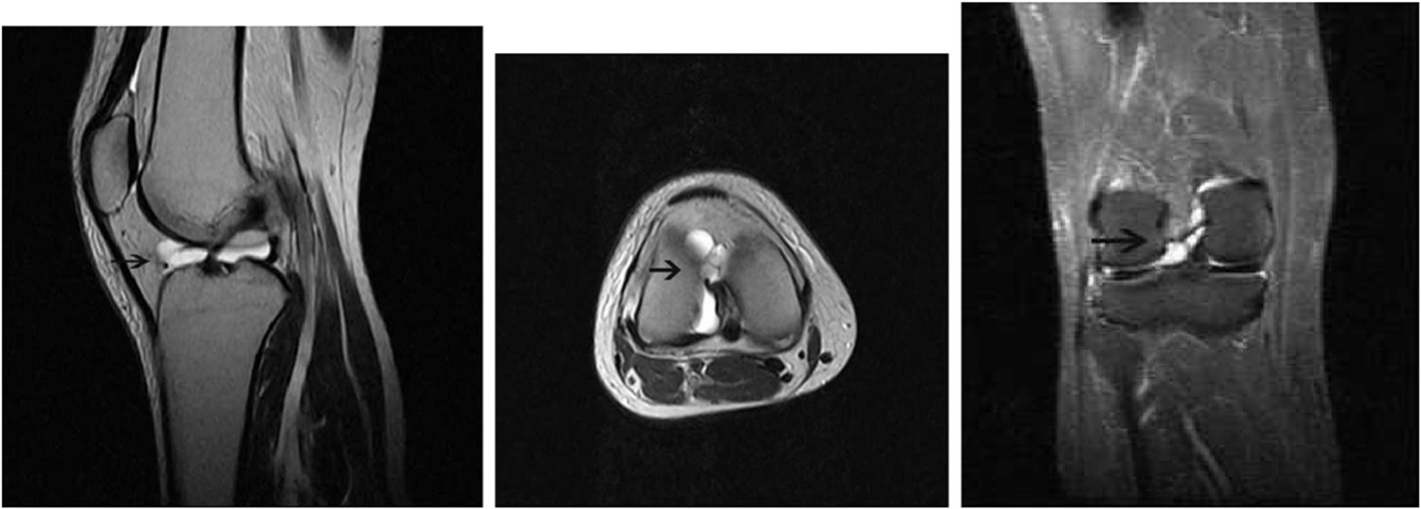
T2-weighted MRI images of the right knee show the multi-lobulated cyst arising from the anterior horn of the medial meniscus to the lateral region of anterior cruciate ligament (black arrow).

An MRI revealed a grade 3 horizontal tear, according to Mink’s classification of the anterior segment of the medial meniscus, and a multi-lobulated cyst arising from the anterior horn of the medial meniscus to the lateral region of the anterior cruciate ligament (ACL) (Figure 2).

**Figure 3:**
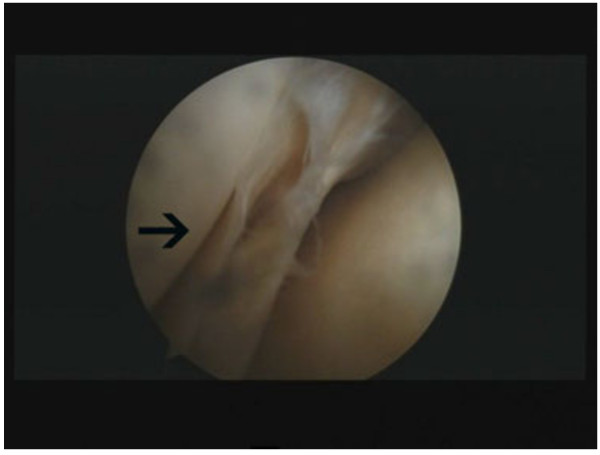
A horizontal tear was located in the anterior segment of the medial meniscus (black arrow).

**Figure 4:**
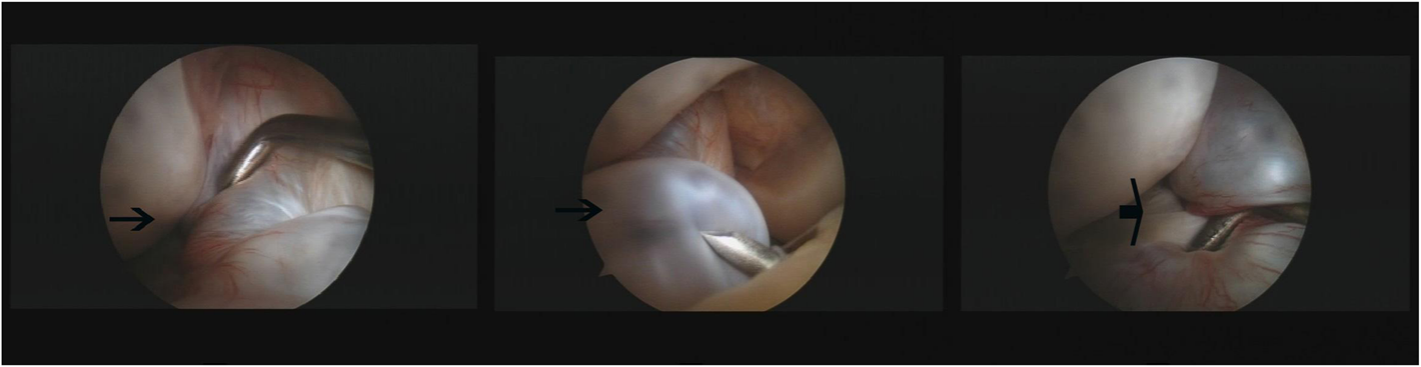
A cyst arising from the anterior horn of the medial meniscus to the lateral region of anterior cruciate ligament (black arrow).

Arthroscopic surgery was performed under general anesthesia and a tourniquet was used. We shaved the cyst only without ACL reconstruction, and at the same time, we repaired the tear in the meniscus. The knee was flexed at a 90 degree angle, on the operating table, using a foot stopper. The ACL, PCL, and also both lateral and medial menisci were intact under arthroscopic examination. There was no evidence of neoplasia, and arthroscopy revealed both medial menisci were intact and cartilage was normal. A horizontal tear was located in the anterior segment of the medial meniscus (Figure 3) and a cyst arose from the anterior horn of the medial meniscus to the lateral region of the ACL (Figure 4).

**Figure 5:**
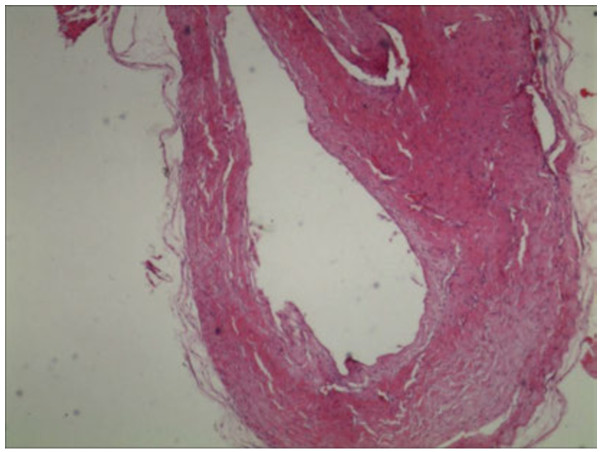
**Histologic section of cyst material showing the ganglion cyst.** (Stain, hematoxylin and eosin, original magnification × 20).

A histological examination of the resected cyst wall, by three pathologists, confirmed the cyst diagnosis. On hematoxylin-and-eosin staining, we can found a fibrous-walled, ganglion cyst associated with fatty cells (Figure 5).

Following surgery, the patient had an uneventful postoperative period, was able to return to her job within 3 weeks, and had no complaints. After the operation, the active range of movement was −0°-120°, and an MRI examination 6 months post-operative revealed that the ACL and meniscal cyst had disappeared. The patient’s pain also disappeared completely during the 3-year follow-up period.

## Discussion

There are several reports of symptomatic ganglion cysts near the anterior cruciate ligament, posterior cruciate ligament, and lateral and medial menisci [8,9]. In particular, we did not find any reports of a symptomatic ganglion originating from the anterior root of the MM to the ACL.

The aetiology of ganglion cysts is unknown. Mucoid degeneration of collagen and connective tissues is widely used to explain the formation of cysts. In addition,trauma or tissue irritation, herniation of the synovium, displacement of synovial tissue during embryogenesis, and proliferating pluripotential messenchymal cells are often thought to cause the cysts [10]. Bergin et al. report that ACL ganglia and mucoid degeneration commonly coexist, and gave some evidence to suggest these two entities may share a similar pathogenesis [11]. In our case, the excised specimen was derived from the MM and ACL, and the cyst had the same organizational structure as a ganglion cyst. We also observed the degradation of meniscus tissue, suggesting that the mucoid degeneration and chronic trauma as a result of the meniscus tear could possibly explain the origin of our cyst.

Occasionally, the clinical manifestation of a cyst is largely dependent on the pathologic process involved, along with its location, size, mass effect, and relationship to surrounding structures [12]. In this case, a preoperative diagnosis was difficult due to a lack of clear signs and symptoms leading to this disease. Therefore, we required that an MRI and arthroscopy be undertaken [13-15]. A diminished range of movement (ROM) is often described as a typical clinical manifestation. In our case, the patent revealed an active range of movement of −10°-100°, and a passive range of movement of −5°-110°. We removed the torn area of the meniscus by arthroscopic surgery, and restored full ROM.

The anterior horn of the MM is attached to the side of ACL anatomically, and provides the anatomical basis for the emergence of this special cyst. Including cyst impingement between the PCL and MM, there have been no reports regarding the primitive appearance, location, and sequence of the cyst, whether cyst initiation happens in the ACL whether it invades in the anterior root of the MM, or on the contrary. In our case, the patient had a horizontal tear located in the anterior segment of the medial meniscus. Relationships exist between cysts and meniscus tears. Barrie [16] found that the menisci cyst was associated with tears, either primarily horizontal or with a horizontal component. Tracks were often demonstrable leading from the tear to the cyst. The relationship of cysts to “myxoid” change of the meniscus is discussed. This suggests that in our instance, the ganglion cyst is maybe produced first at the anterior horn of the MM, then incurs into the ACL. And surgical repair of the meniscus avulsion and restoration of the anatomy and biomechanical function of menisci as early as possible will reduce the ACL lesion of this kind of cyst.

## Conclusion

The ganglion cyst on the ACL can be caused by injury of the anterior horn of the meniscus and on the premise of guarantee the stability of the knee joint, A surgery limited on the meniscus can solve the problem in the early time.

## Consent

Written informed consent was obtained from the patient for publication of this case report and any accompanying image. A copy of the written consent is available for review by the Editor-in-Chief of this journal.

## Competing interests

The authors declare that they have no competing interests.

## Authors’ contributions

MG and HY carried out the surgical treatment, GZ discussed the results and commented on the manuscript. All authors have read and approved the final manuscript.

## Pre-publication history

The pre-publication history for this paper can be accessed here:

http://www.biomedcentral.com/2052-1847/5/22/prepub
